# Household preferences for pet keeping: Findings from a rural district of Sri Lanka

**DOI:** 10.1371/journal.pone.0277108

**Published:** 2022-11-22

**Authors:** Devarajan Rathish, Jayanthe Rajapakse, Kosala Weerakoon

**Affiliations:** 1 Department of Veterinary Pathobiology, Faculty of Veterinary Medicine and Animal Science, University of Peradeniya, Peradeniya, Sri Lanka; 2 Department of Family Medicine, Faculty of Medicine and Allied Sciences, Rajarata University of Sri Lanka, Saliyapura, Sri Lanka; 3 Department of Parasitology, Faculty of Medicine and Allied Sciences, Rajarata University of Sri Lanka, Saliyapura, Sri Lanka; Tufts University Cummings School of Veterinary Medicine: Cummings School of Veterinary Medicine at Tufts University, UNITED STATES

## Abstract

Pet ownership is an integral part of a modern-day family. It provides a wide range of benefits to humans. However, data on pet ownership are relatively limited from rural regions, Southern Asia and low-middle-income countries. We aim to report the prevalence and associated factors for pet ownership and veterinary visits in Anuradhapura, Sri Lanka. A community-based, cross-sectional study was conducted. An interviewer-administered questionnaire was used. Binary logistic regression was performed to determine significant associations between variables of interest and pet ownership (p < 0.05). Out of the 532 households, 57% currently owned a pet. The most common pet was the dog owned by 41% of the households and the cat was the second most owned by 17%. Security (69% - 152/220) was the most common role for dogs at home while it was companionship for cats (31% - 27/88) and hobby for both birds (64% - 18/28) and fish (54% - 14/26). Most dogs (54% - 118/220) had one veterinary visit within the last year. Households with >1 adult female [p = 0.02; OR = 1.61 (95% CI 1.09 to 2.36)], participants living alone [p = 0.03; OR = 0.24 (95% CI 0.07 to 0.86)] and Buddhists [p = 0.02; OR = 2.56 (95% CI 1.16 to 5.63)] were significantly associated with pet ownership. Pet ownership is common among people in Anuradhapura, Sri Lanka, with a few demographic factors having a significant association with pet ownership. Dogs are the most common type of pet and highlight the opportunity for research related to canine companionship and human health. Future research on such topics should consider the above-mentioned socio-demographic predictors as potential confounders.

## Introduction

In modern days, companionship is a common reason for having pets, and pet owners consider their pets as valued family members [1]. Pet animals influence the family throughout life [2]. Pet ownership contributes to healthy relationships with neighbours [3] and improved physical and mental health via social support [3]. Well-controlled experimental studies have revealed human physical health benefits related to human-animal interactions [4–6]. Pet ownership contributes to improved mental health via enhancing companionship, reducing loneliness and increasing socialization among elders [7]. However, pet ownership also involves high costs, injuries, damage to property, zoonosis, grief following death and adverse social events [8, 9].

A global study done in 22 countries showed that more than half of the people around the globe have a pet in their homes with the dog being the most common pet (33%). Cats and fish were the second and third most popular pet with ownership at 23% and 12% respectively [10]. Accordingly, Argentina had the highest incidence of pet ownership followed by Mexico and Brazil. Dog ownership in the above-mentioned countries was 66%, 64% and 58% respectively. However, pet ownership varies widely around the world. However, Turkey (12%), Hong Kong (14%) and Japan (17%) recorded the lowest percentage of dog ownership [10]. A similar study revealed that 59% of the people in Asia had a pet; the ownership ranged from 28% in Japan to 83% in the Philippines [11]. And, dogs (32%) were once again the most popular pets followed by cats (26%). In India, the neighbouring country of Sri Lanka, pet ownership and dog ownership was reported to be 59% and 34% respectively [11]. Households were more likely to own a dog if they had an adult female household member [12]. However, pet ownership was significantly lower among those who lived alone [13].

Globally, an integrative effort has been made across multiple scientific fields toward One Health that could bring together human and veterinary medicine [14]. Human-animal interaction has been explored to improve both human and animal health [15]. One Health focuses on the family’s health and well-being by identifying the role of companion animals [16]. Pet ownership, animal-assisted activity, animal-assisted therapy, and service animals are contexts in which human-companion animal interaction takes place. Therefore, it is essential to have background information on pet ownership to implement One Health via human-animal interaction [17]. Most of the studies on the above concepts are reported from the developed world [18]. Data on pet ownership are relatively limited from the rural regions, Southern Asia and low-middle-income countries. There are few studies on dog ownership from urban regions of India [19] and Sri Lanka [20]. Rural communities have shown a higher rate of pet and dog ownership than urban communities, suggesting the potential for beneficial impacts of human-companion animal interactions [21].

Anuradhapura is a rural, agrarian district of Sri Lanka, where 94% belongs to the rural sector and 51% of the employment belongs to agriculture [22, 23]. Therefore, the population mostly dwell near its household in rural and agrarian districts and have tight-knit communities. Also, Anuradhapura is the largest district by surface area in Sri Lanka. However, the population density of the district is much lower compared to the country’s density [22]. Hence, there is more space per household in Anuradhapura when compared to urbanized districts. The above facts show that Anuradhapura is a good representation of a rural region that can provide insights into low-middle-income, Southern Asian countries. Further, information on pet ownership at the community level would help identify trends in pet services, frame public strategy, plan animal sheltering and organize animal welfare programmes [21, 24]. Moreover, identifying socio-demographic predictors associated with pet ownership would help consider potential confounders in One Health initiative and human-animal interaction studies. We aim to report the prevalence and associated factors for pet ownership and veterinary visits in Anuradhapura, Sri Lanka.

## Materials and methods

### Study design, setting and population

A community-based, cross-sectional survey design was used. The study was conducted in the Anuradhapura district. Anuradhapura district is a rural [22] and agrarian [23] district which is the largest by surface area in Sri Lanka. The dwellers in the district during the study period were considered the study population.

### Sampling size

The minimum sample size was calculated using the equation of n = [Z^2^xP(1-P)]/d^2^ [25]. Where n is the sample size (384), Z is the Z statistic for a level of confidence (1.96), P is the expected prevalence or proportion (0.5), and d is the precision of the estimate (0.05). After calculating with a design effect of 1.25, 480 was achieved. With an addition of 10% for non-response, a minimum of 528 households must be recruited from the district.

### Selection criteria and sampling method

Households with at least one adult aged ≥ 18 years and residing in Anuradhapura for ≥ 5 years were included. Households were selected to represent each of the 22 divisional secretariats of the Anuradhapura district using probability proportional to size. The grama niladhari division with the highest population for each divisional secretariat was selected for data collection. After rounding to the last decimal, a total of 532 households needed to be recruited. The Google map point of the selected grama niladhari division was considered as the starting point. The household nearest to the starting point was the first to be included. A road or a path was followed to identify the next households until the expected number of households was selected from each divisional secretariat division. Households were approached sequentially, one after the other. Hence, the sample can be considered representative of a larger population. No data was collected from households that refused to participate. Also, if no one was at home, the next household was approached.

### Study instruments

An interviewer-administered questionnaire was used to collect data (S1 File). The questionnaire had two separate parts: socio-demographic factors and pet ownership. The following socio-demographic factors were considered: age, years residing in Anuradhapura, divisional secretariat division, whether the participant was the head of household, sex, education level, employment, employment type, marital status, sector, house structure, whether the participant was living alone, number of adults and children at home (males and females), religion, family income, and whether every adult household member was employed full time. And, the following details regarding pet ownership were considered: number of pets, years of pet ownership, the role of pet, source of pet, type of pet food, where commercial food was bought for pets, living area of pet, other special influence by pets on the family, Veterinary visits and rabies vaccination. The questionnaire was drafted by the first author by reviewing relevant previous literature on similar studies [13, 26–30] and critically revised by the other authors. The questionnaire was translated into two local languages (Sinhala and Tamil). The translated questionnaire was pre-tested in a sample of residents in the Anuradhapura district. The interviewer-administered questionnaire was finalized following minor amendments including the order of the questions.

### Data collection

Ethical clearance was obtained from the Ethics Review Committee of the Faculty of Medicine and Allied Sciences, Rajarata University of Sri Lanka (ERC/2020/76). Prior permission was obtained from the Regional Director of Health Services, Anuradhapura. Further, all Medical Officers of Health in the Anuradhapura district were informed by the Regional Director of Health Services to facilitate data collection in their areas. The selected households were briefed about the study and its benefit to the community. Informed verbal consent following an explanation of the study was considered suitable as the study does not collect any sensitive data nor does it involve any participants below the age of 18 years, anthropometric measurements or clinical sampling. None of the participants was requested to show the pet rabies vaccination cards to avoid any degree of coercion. Upon the informed verbal consent, the above-mentioned questionnaire was administered to the most senior adult occupant consenting for the interview. Each interview lasted for around 10 to 15 minutes. Explaining the study, obtaining verbal consent and data collection was done by the principal investigator and trained data collectors who have passed the General Certificate of Education (Advanced Level) Examination, Sri Lanka. Training of the data collectors was done by the principal investigator. All necessary precautions were taken concerning COVID-19 during the data collection. Data collections of 9 divisional secretariat divisions were completed in April 2021, before the travel restrictions for the 3^rd^ peak of COVID-19 cases in Sri Lanka. Data collections of the remaining divisional secretariat divisions were completed in July 2021, after the travel restrictions were lifted.

### Data description and analysis

The prevalence of pet ownership was computed as a point estimate with a 95% confidence interval. Categorical variables were presented as frequencies with percentages while continuous variables were presented as means with standard deviation. The analysis was performed using Microsoft Excel and add-ins. Univariable analyses were performed using a chi-square test to identify possible associations for the variables of interest. Variables with a p < 0.2 were included in a multivariable model. A backward stepwise regression was used to identify variables with a p < 0.05. Also, a binary logistic regression was performed including all variables of interest against the pet ownership (odds ratios with 95% CI).

### Definitions

- Head of household—*“Head of a household is the person who usually resides in the household and is acknowledged by the other members of the household as the head”* [31].
- Pet ownership—A household that *“privately-owns companion animal(s) not intended for research or resale”* [32] as declared by the participants.
- Rural sector—*“All areas other than urban and estate”* [31].
- Urban sector—*“All areas administered by Municipal and Urban councils”* [31].

## Results

### Demographic data

A total of 547 households were approached and data from a total of 532 households were collected (97%). There was no one at home in 11 households on the day of data collection. And, members of 04 households refused participation as they were busy with their day-to-day activities. The mean age of the participants was 38.2 (SD 16.1) years. The mean duration of stay at Anuradhapura was 45.4 (SD 14.1) years. Most of the study participants were non-head of household (54% - 286/532), females (56% - 295/532), Buddhists (94% - 500/532), educated up to or above general certificate of education (ordinary level) (78% - 415/532), employed (52% - 275/532), full-time employed (96% - 264/275), married (84% - 449/532), rural residents (92% - 489/532) and not living alone at the household (96% - 513/532). Also, most had single storied-single house (93% - 492/532), ≥1 adult male in the household (96% - 513/532), ≥1 adult female in the household (98% - 519/532), no male children in the household (76% - 402/532), no female children in the household (64% - 342/532) and a monthly household income of less than 50,000 Sri Lankan rupees (66% - 353/532). Further, only 7% (39/532) had every member ≥18 years employed full-time.

### Pet ownership

**Fig 1:**
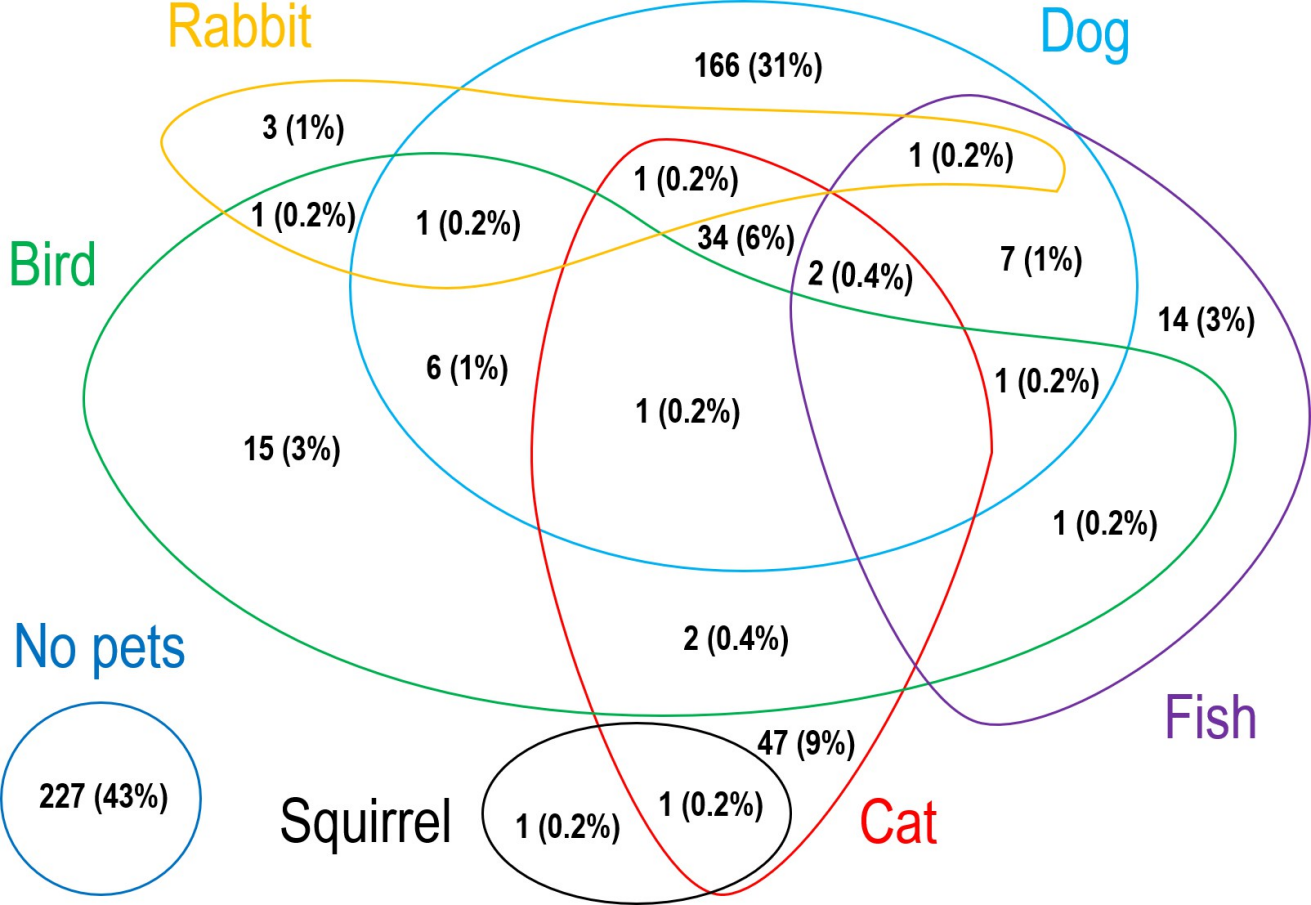
Reasons for not owning a pet.

**Fig 2:**
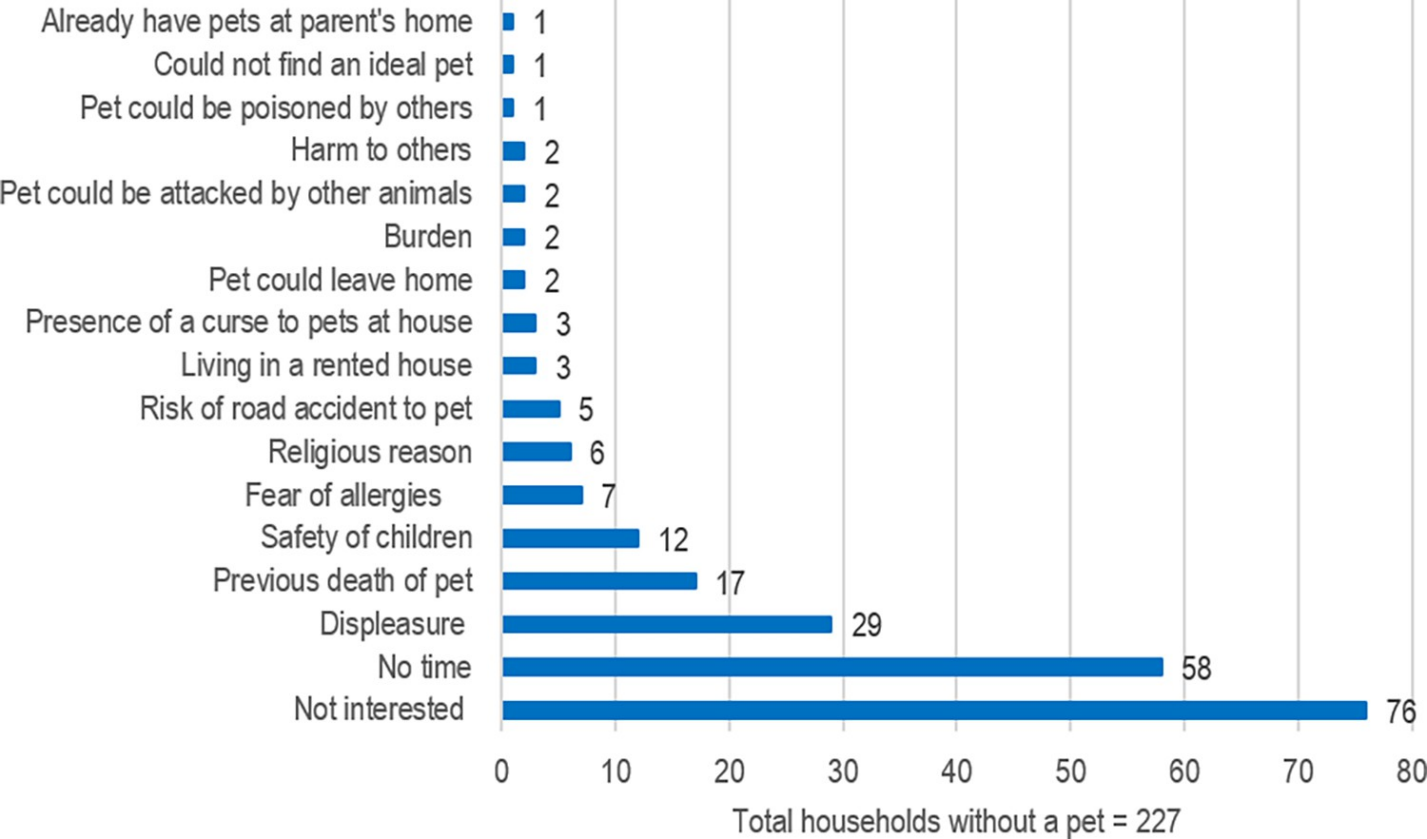
Number and percentage of pet combinations in the selected households (n = 532).

Out of the selected households, 57% (305/532; 95% CI 53–62) currently owned a pet (Table 1). Fifteen per cent (77/532) did not currently own a pet but had one previously and 28% (150/532) never owned a pet. Out of those who did not currently own a pet, most (34% - 76/227) had mentioned a lack of interest as the reason for not owning a pet (Fig 1). The dog was owned as a pet in 41% (220/532) of the households, cat in 17% (88/532), bird in 5% (28/532), fish in 5% (26/532) and rabbit in 1% (7/532). The squirrel was found only in two households. The dog was the only pet in 31% (166/532) of the households while both dogs and cats alone were seen in 6% (Fig 2). Five per cent (25/532) owned non-pet animals. The duration of non-pet animal ownership ranged from 1 to 20 years. The cow was owned in 13 households (1.5 to 20 years) followed by hens in 8 (1 to 10 years), goats in 5 (3 to 10 years) and ducks in 2 (1 to 5 years).

**Table 1:** Percentage of households with different types of pets by divisional secretariat division.

No	Divisional secretariat division	Total number of households	Percentage (%) of households with a						
			Pet	Dog	Cat	Fish	Bird	Rabbit	Squirrel
1	Galenbidunuwewa	29	28	24	0	7	0	0	0
2	Galnewa	21	67	43	19	10	14	0	0
3	Horowpothana	23	65	35	39	4	13	4	0
4	Ipalogama	24	38	21	8	8	0	0	0
5	Kahatagasdigiliya	25	28	24	8	0	0	0	4
6	Kebithigollewa	14	71	50	7	21	7	0	0
7	Kekirawa	36	64	50	19	8	3	3	0
8	Mahavilachchiya	14	64	57	14	0	7	0	0
9	Medawachchiya	29	41	31	14	3	0	0	0
10	Mihintale	22	73	64	18	0	0	0	0
11	Nachchaduwa	16	56	38	25	0	0	0	0
12	Nochchiyagama	31	52	42	23	0	0	0	0
13	Nuwaragam Palatha Central	38	63	29	13	13	18	3	0
14	Nuwaragam Palatha East	43	72	40	21	5	12	2	2
15	Padaviya	14	100	93	36	0	0	0	0
16	Palagala	21	71	48	14	5	10	0	0
17	Palugaswewa	10	30	10	20	0	0	0	0
18	Rajanganaya	21	62	48	24	0	0	0	0
19	Rambewa	23	48	30	17	0	0	0	0
20	Thalawa	35	69	66	9	11	9	6	0
21	Thambuththegama	26	35	27	8	0	8	4	0
22	Thirappane	17	76	65	24	0	0	0	0
	**Total**	**532**							

Most dog (77% - 169/220) and cat owners (88% - 78/88) owned only one in number while most bird (86% - 24/28) and fish owners (96% - 25/26) owned three or more. Most dog (62% - 136/220) and cat owners (51% - 45/88) owned their pet for > 1 to ≤ 5 years while most bird (75% - 21/28) and fish owners (69% - 18/26) owned their pets for ≤ 1 year. Security (69% - 152/220) was the most common role for dogs at home while it was companionship for cats (31% - 27/88) and hobby for both birds (64% - 18/28) and fish (54% - 14/26). Most dogs were gifted or inherited (49% - 109/220) while cats were owned when strayed (59% - 52/88). The birds (93% - 26/28) and fish (92% - 24/26) were bought from a pet store. Home-cooked food was the most common for dogs (78% - 172/220) and cats (74% - 65/88) while it was commercial dry food for birds (93% - 26/28) and fish (92% - 24/26). Most commercial foods were bought from supermarkets for dogs (46% - 6/13) and cats (100% - 1/1) while it was from the variety store for birds (63% - 12/19) and fish (60% - 15/25). Most dogs (68% - 149/220) and cats (52% - 46/88) lived outdoor in a non-specific area while most birds (82% - 23/28) and fish (61% - 16/26) lived outdoor in a specific area. Moreover, dogs (13% - 29/220), cats (6% - 5/88) and birds (39% - 11/28) reduced travel of the family (Table 2).

**Table 2:** Demography of pet ownership.

Variables	Dog	Cat	Bird	Fish	Rabbit	Squirrel
***A*. *Owners (n)***	*220*	*88*	*28*	*26*	*7*	*2*
***B*. *Number of pets (%)***						
1	169 (77)	78 (88)	1 (3)	1 (4)	2 (29)	2 (100)
2	45 (20)	5 (6)	3 (11)	0	3 (43)	0
≥3	6 (3)	5 (6)	24 (86)	25 (96)	2 (29)	0
***C*. *Years of ownership (%)***						
≤ 1	37 (17)	38 (43)	21 (75)	18 (69)	5 (71)	1 (50)
> 1 to ≤ 5	136 (62)	45 (51)	7 (25)	6 (23)	2 (29)	1 (50)
> 5 to ≤ 10	43 (19)	5 (6)	0	2 (8)	0	0
> 10	4 (2)	0	0	0	0	0
***D*. *Role of pet (%)*** *						
Companionship	42 (19)	27 (31)	6 (21)	4 (15)	1 (14)	1 (50)
Education for children	3 (1)	1 (1)	0	0	0	0
Exercise	0	2 (2)	0	0	0	0
Fun for children	52 (24)	15 (17)	7 (25)	8 (31)	4 (57)	0
Hobby	26 (12)	19 (22)	18 (64)	14 (54)	5 (71)	1 (50)
Pet gifted or inherited to household no additional role	26 (12)	8 (9)	0	0	0	0
Pet received as stray to household no additional role	40 (18)	22 (25)	0	0	0	0
Protection against rats	0	8 (9)	0	0	0	0
Responsibility for children	1 (1)	0	0	0	0	0
Relaxation	1 (1)	0	0	2 (8)	0	0
Security	152 (69)	5 (6)	0	0	0	0
Work	3 (1)	0	0	0	0	0
***E*. *Source of pet (%)***						
Animal shelter	2 (1)	0	0	0	0	0
Gift or inherited	109 (49)	35 (40)	2 (7)	2 (8)	1 (14)	0
Pet store	28 (13)	1 (1)	26 (93)	24 (92)	6 (86)	0
Stray	81 (37)	52 (59)	0	0	0	2 (100)
***F*. *Type of pet food (%)*** *						
Commercial chilled	3 (1)	1 (1)	0	0	1 (14)	0
Commercial dry	7 (3)	0	19 (68)	25 (96)	2 (29)	0
Commercial wet	3 (1)	0	0	0	1 (14)	0
Home grains	0	0	1 (4)	0	0	0
Home-cooked	172 (78)	65 (74)	4 (14)	1 (4)	0	2 (100)
Raw	4 (2)	3 (3)	0	0	3 (43)	0
Scraps	71 (32)	34 (39)	12 (43)	0	3 (43)	0
***G*. *Where commercial food was bought for pets (%)*** * ^						
Online	1 (8)	0	0	0	0	0
Pet store	0	0	4 (21)	6 (24)	1 (50)	0
Rural supplier	1 (8)	0	4 (21)	6 (24)	0	0
Supermarket	6 (46)	1 (100)	0	1 (4)	0	0
Variety store	5 (38)	0	12 (63)	15 (60)	1 (50)	0
***H*. *Living area of pet (%)***						
Indoor specific area	4 (2)	1 (1)	4 (14)	8 (31)	0	2 (100)
Indoor no specific area	11 (5)	40 (46)	0	0	1 (14)	0
Outdoor specific area	56 (25)	1 (1)	23 (82)	16 (61)	6 (86)	0
Outdoor no specific area	149 (68)	46 (52)	1 (4)	2 (8)	0	0
***I*. *Other special influence by pets on the family (%)*** *						
Better health status	2 (1)	1 (1)	0	0	0	0
Socially active	21 (10)	2 (2)	2 (7)	0	1 (14)	0
Activities delayed or reduced	4 (2)	2 (2)	0	0	0	0
Economic burden	7 (3)	0	0	0	0	0
Health issues related to kids	0	1 (1)	0	0	0	0
Hygienic issues	1 (1)	4 (5)	0	0	0	0
Impaired relationship with neighbours/visitors	4 (2)	0	0	0	0	0
Increased workload	5 (2)	0	2 (7)	0	0	0
Injuries by pet	17 (8)	5 (6)	0	0	0	0
More stray dogs and cats entering the village	1 (1)	1 (1)	0	0	0	0
Reduced travel	29 (13)	5 (6)	11 (39)	0	3 (43)	0

* Participants were allowed to select multiple options

^ Among participants that fed commercial food

### Veterinary visits and rabies vaccination

Most dogs (67% - 148/220) had at least one veterinary visit within the last year. However, most cats (60% - 53/88), birds (93% - 26/28) and fish (100% - 26/26) had no veterinary visit within the last year. Vaccination was the most common reason for a veterinary visit for dogs (97% - 144/148) and cats (91% - 32/35) while health issue was the reason for birds (100% - 2/2). Most of those who did not take their pet dog (79% - 57/72), cat (91% - 48/53), bird (100% - 26/26) and fish (100% - 26/26) for a veterinary visit said it was not necessary during the last year (Table 3). Out of the houses which owned a dog, 90% (197/220) had vaccinated their pet within the last year while it was 50% for cats. Out of the houses which owned both dogs and cats, 61% (23/38) had vaccinated both their pets within the last year while 34% (13/38) had vaccinated only the dog and 5% (2/38) had not vaccinated both their pets.

**Table 3:** Demography of veterinary visits.

Variables	Dog	Cat	Bird	Fish	Rabbit	Squirrel
***A*. *Owners (n)***	***220***	***88***	***28***	***26***	***7***	***2***
***B*. *Number of veterinary visits within the last year (%)***						
None	72 (33)	53 (60)	26 (93)	26 (100)	6 (86)	2 (100)
1	118 (54)	31 (35)	2 (7)	0	0	0
> 1 to ≤ 3	18 (8)	4 (5)	0	0	1 (14)	0
> 3	12 (5)	0	0	0	0	0
***C*. *Reasons for veterinary visits (%)*** * ^						
Annual check	18 (12)	2 (6)	0	0	1 (100)	0
Desexing	8 (5)	2 (6)	0	0	0	0
Emergency	1 (1)	0	0	0	0	0
Health issue	31 (21)	4 (11)	2 (100)	0	1 (100)	0
Vaccination	144 (97)	32 (91)	0	0	1 (100)	0
***D*. *Reason not taken to a veterinary visit (%)*** * ^#^						
Accessibility	10 (14)	4 (8)	0	0	0	0
Cost	2 (3)	1 (2)	0	0	0	0
Forgot to give the vaccination	0	1 (2)	0	0	0	0
Not necessary	57 (79)	48 (91)	26 (100)	26 (100)	6 (100)	2 (100)
No vet facility nearby	3 (4)	2 (4)	0	0	0	0
Vaccination at temple	3 (4)	0	0	0	0	0

* Participants were allowed to select multiple options

^ Among owners reporting ≥1 visit to a veterinarian

^#^ Among owners who did not take their pet to a veterinarian

### Factors associated with pet ownership

The backward stepwise regression found households with >1 adult female, participants living alone and Buddhists to have a p < 0.05. The Cox-Snell R^2^ of the three variable regression was 0.05 with the following predictive model:

Pet ownership = - 0.84 (intercept) + 0.54 (households with >1 adult females) - 1.38 (participants living alone) + 0.97 (Buddhists)

The binary logistic regression (Cox-Snell R^2^ = 0.07) for variables of interest against pet ownership revealed the above same three variables to have a p < 0.05. Accordingly, households with >1 adult female [p = 0.02; OR = 1.61 (95% CI 1.09 to 2.36)], participants living alone [p = 0.03; OR = 0.24 (95% CI 0.07 to 0.86)] and Buddhists [p = 0.02; OR = 2.56 (95% CI 1.16 to 5.63)] were significantly associated with pet ownership. Out of the households with >1 adult female, 65% (173/266) owned a pet compared to 50% (132/266) in households with ≤1 adult female. Out of the participants living alone, 21% (4/19) owned a pet compared to 59% (301/513) among those who did not live alone. Among Buddhists, 59% (294/500) owned a pet compared to 34% (11/32) of non-Buddhists. There was no significant association between pet ownership and other variables of interest (Table 4). Further, a binary logistic regression for variables of interest against dog ownership revealed that households with >1 adult female [p = 0.01; OR = 1.69 (95% CI 1.15 to 2.48)] and Buddhists [p = 0.01; OR = 4.16 (95% CI 1.54 to 11.29)] were significantly associated with dog ownership. Moreover, households with >1 adult male [p = <0.01; OR = 5.73 (95% CI 1.95 to 16.84)], households with ≥ 1 female child [p = 0.047; OR = 2.55 (95% CI 1.01 to 6.44)], living in urban sector [p = 0.03; OR = 4.07 (95% CI 1.15 to 14.48)] and Buddhists [p = 0.02; OR = 0.24 (95% CI 0.07 to 0.81)] were significantly associated with bird ownership. However, cat and fish ownership failed to have a significant association with any of the variables of interest.

**Table 4:** Binary logistic regression for the variables of interest against pet ownership.

*Variable*	*Description*	*Pet ownership*		*Coefficient*	*p-value*	*Odds ratio*	*95% Confidence interval*	
		*Yes*	*No*				*Lower*	*Upper*
1. Years living in Anuradhapura -	> 40 years	126	92	0.16	0.54	1.18	0.70	1.99
	≤ 40 years	179	135					
2. Age	≤ 40 years	126	96	0.25	0.40	1.28	0.72	2.28
	> 40 years	179	131					
3. Participant	Head of household	138	108	0.15	0.58	1.16	0.69	1.96
	Other household members	167	119					
4. Sex	Male	134	103	-0.20	0.46	0.82	0.48	1.39
	Female	171	124					
5. Education	≤ Primary	61	56	-0.12	0.64	0.89	0.55	1.44
	> Primary	244	171					
6. Employment	Employed	158	115	-0.57	0.42	0.56	0.14	2.29
	Unemployed	147	112					
7. Employment type	Full time	154	110	0.63	0.37	1.88	0.47	7.60
	Part-time or unemployed	151	117					
8. Marriage	Registered or customary married	259	190	-0.06	0.83	0.94	0.53	1.66
	Never married, widowed, divorced and separated (legally or not legally)	46	37					
9. No adult males (≥12 years)	> 1	163	99	0.27	0.18	1.31	0.89	1.95
	1	142	128					
*10*. *No adult females (≥12 years)*	*> 1*	*173*	*93*	*0*.*47*	*0*.*02*	*1*.*61*	*1*.*09*	*2*.*36*
	*1*	*132*	*134*					
11. No male children (<12 years)	≥ 1	69	61	-0.21	0.35	0.81	0.53	1.26
	None	236	166					
12. No female children (<12 years)	≥ 1	102	88	-0.18	0.39	0.83	0.55	1.26
	None	203	139					
13. Sector	Urban	31	12	0.57	0.13	1.77	0.84	3.73
	Rural	274	215					
14. House structure	Single house single storied	280	212	-0.18	0.63	0.84	0.41	1.72
	Other	25	15					
*15*. *Living alone*	*Yes*	*4*	*15*	*-1*.*44*	*0*.*03*	*0*.*24*	*0*.*07*	*0*.*86*
	*No*	*301*	*212*					
*16*. *Religion*	*Buddhism*	*294*	*206*	*0*.*94*	*0*.*02*	*2*.*56*	*1*.*16*	*5*.*63*
	*Other*	*11*	*21*					
17. Monthly household income	< Rs 50,000	201	152	0.15	0.47	1.17	0.77	1.76
	≥ Rs 50,000	104	75					
18. Every member ≥18 years employed full-time	Yes	18	21	-0.01	0.97	0.99	0.47	2.08
	No	287	206					
19. Ownership of non-pet animals	Yes	12	13	-0.33	0.45	0.72	0.31	1.70
	No	293	214	0.16				

Italic values indicate significance with a p-value of < 0.05

## Discussion

The present study, to the best of our knowledge, is the first overview of the prevalence and associated factors for pet ownership, veterinary visits and rabies vaccination in rural Sri Lanka. The findings would be a vital guide in health care and future research related to one-health among people of low-middle income and rural regions. The study reported that more than half of the participants owned a pet in the Anuradhapura district. Dogs were the most common pet and were well vaccinated against rabies within the last year. Cats were placed second but only half of its population were vaccinated against rabies. Most dog and cat owners owned their pets for 1 to 5 years while most bird and fish owners owned their pets for ≤ 1 year. The above years of ownership may correlate with the general average lifespan of the relevant animals. Households having more than one adult female, participants living alone and Buddhists were significantly associated with pet ownership. However, a study in the United Kingdom found pet ownership in childhood and adolescence to be associated with sex, birth order, maternal age, maternal education, number of people in the household, house type, and concurrent ownership of other pets [33].

Similar to the present study, more than half of the people in the globe [10] and Asia [11] have a pet in their homes. Also, the dog has been the most common pet both in the globe (33%) [10] and in Asia (32%) [11]. Our study reported a higher percentage (41%) of households in Anuradhapura that own a dog. In India, the neighbouring country of Sri Lanka with similar culture and climate, pet ownership and dog ownership were seen at 59% and 34% respectively [11]. In contrast, cats were the most common pet in New Zealand which is quite different in culture and climate to Sri Lanka [27]. Thailand, a country where the vast majority of people follow Buddhism, reported a similar percentage of dog owners (47%). It is interesting to note that our study found Buddhists to be significantly associated with both pet and dog ownership. Sri Lanka is a country with cultural and climate diversity. Therefore, future similar studies in Sri Lanka are proposed to involve participants from different climates and cultures. In the present study, the most common reason for owning a dog was security, which was similar to the Philippines [34] and contrary to companionship in New Zealand [27]. Future studies are needed to find out if the economic status of a country or a community would influence the reason for owning a dog. As in prior literature [27, 35], lack of interest was the most common reason for not owning a pet. Households from a community in the United Kingdom were more likely to own a dog if they had an adult female household member [12]. Our study also showed that households with more than one adult female were more likely to own a pet and a dog. The United States of America reports that pet ownership was significantly lower among those who lived alone which was in line with the findings of the present study [13]. Therefore, future One Health initiatives could consider the presence of adult females and the number of family members.

Raw food for diet is not without risk for both the pet [36] and the owner [37]. The majority of the participants fed their dogs and cats with home-cooked food. Only a very few fed their pet dogs and cats with raw food. It is encouraging to note that people from a rural region had taken precautions in minimizing risks related to raw food consumption. However, providing home-cooked food would have been affordable to the pet owner of a rural community. Also, a previous study in a relatively high-income area of Colombo city of Sri Lanka revealed that most dog owners fed their pets with home-cooked food. Further, dogs consuming commercial food for the majority of their diet were no less likely to receive dietary supplements than dogs fed home-cooked food for the majority of their diet [38]. Moreover, a Brazilian study concludes that commercial wet diets for maintenance dogs were the most expensive for 1000 kcal of metabolizable energy followed by home-cooked and commercial dry diets [39].

Most of the dogs in the present study had a veterinary visit and were vaccinated against rabies within the last year. The above findings highlight responsible dog ownership. Responsible pet ownership includes: *“Protecting pets from pain*, *suffering*, *injury and disease and registering with a veterinary practice and implementing their advice regarding the preventive and therapeutic healthcare needs of your pet”* [40]. Rabies is a vaccine-preventable but fatal zoonotic, viral disease and dogs are the main source of human rabies transmission and deaths [41]. Sri Lanka has depended mainly on post-exposure prophylaxis and dog vaccination to achieve its goal of rabies elimination [42]. Annual rabies vaccination of all dogs above six weeks is recommended by Sri Lankan rabies control authorities [43]. The findings of our study furnish evidence for excellent progress. Nevertheless, only half of the feline population was vaccinated against rabies within the last year. Information and education campaign materials could be used to enhance the knowledge, attitude and practice of rabies in rural Sri Lanka [44]. Although most of the dogs had a veterinary visit within the last year, most cats, birds and fish did not visit a veterinarian as participants did not consider it necessary. Similar to the present study, the most common reason for a veterinary visit was for vaccination or annual check-up in New Zealand [27]. And, the above same reason was given by the vast majority of pet owners for not taking their pets to a veterinarian [27].

The study was limited to a rural district of Sri Lanka which cannot be generalized to the entire country. Also, the findings of a cross-sectional study cannot establish a causal relationship between pet ownership and the associated factors. Further, pet ownership was determined by the declaration of participants and pet bonding was not assessed. Assessment of pet bonding using a pet bond scale could help assess the extent of attachment between the pet and the owner. Subsequently, the level of bonding could be used in analysing associated factors. Moreover, during the present study, if no one was at home, the next household was approached. Retaining a household for recontact if no one was at home is methodologically challenging considering the large surface of the district and pandemic-related travel restrictions. However, only 11 households had no one at home on the day of data collection. And, members of only 04 households refused participation as they were busy with their day-to-day activities. A higher participation rate was possible probably because the population in rural and agrarian districts dwell mostly near its household, the pandemic-related travel restrictions and the short time taken for the interview. Further, the grama niladhari division with the highest population for each divisional secretariat was selected for data collection. However, the prevalence of pet ownership may be different in grama niladhari divisions with lower populations. The inclusion of these areas is methodologically challenging in a study involving an entire district that is the largest by surface area in its country. Separate research on such areas is proposed. Moreover, the data on rabies vaccination within the last year was solely derived from the participant’s verbal information as none of the participants were requested to show the rabies vaccination cards to avoid any degree of coercion. Nevertheless, the findings of the present study are unique because it was conducted in a rural district of a South Asian country where similar studies were scarce.

The World Health Organization defines One Health as “an approach to designing and implementing programmes, policies, legislation and research in which multiple sectors communicate and work together to achieve better public health outcomes”. It identifies the health of humans, domestic animals, and the environment are symbiotic [45]. Also, the Centers for Disease Control and Prevention mentions the human-animal bond as an example of One Health because the bond can help improve mental well-being [14]. The study provides vital data on pet ownership from a rural region of a lower-middle-income, South Asian country. Information on pet ownership at the community level would help identify trends in pet services, frame public strategy, plan animal sheltering and organize animal welfare programmes [21, 24]. The findings will be useful in planning out research and intervention related to One Health involving both human and veterinary medicine. Also, the higher percentage of dog ownership highlights the opportunity for research related to canine companionship and human health. Future research on such topics in One Health initiative should consider the socio-demographic predictors as potential confounders. Further, future research should focus on the assessment of pet bonding and the identification of ways to improve veterinary care among households with a pet. Assessment of pet bonding using a pet bond scale could help assess the extent of attachment between the pet and the owner. Subsequently, the level of bonding could be used in analysing associated factors.

## Conclusions

Pet ownership is common among households of Anuradhapura, Sri Lanka. The number of adult females, participants living alone and Buddhists were significantly associated with pet ownership. Dogs are the most common type of pet and were well vaccinated against rabies within the last year. These findings highlight the opportunity for research related to canine companionship and human health. However, the above-mentioned socio-demographic predictors should be considered as potential confounders in the One Health initiatives. Although most of the dogs had a veterinary visit within the last year, most cats, birds and fish did not have a veterinary visit as the owners considered it unnecessary. While the study findings would contribute toward health care improvement in rural regions, in line with One Health concept, future studies are necessary to identify ways to improve the veterinary care of pets and assess pet bonding.

## Supporting information

S1 File The datasheet of the study. (XLSX)

## Abbreviations

CI: confidence interval
COVID-19: coronavirus disease
OR: odds ratio
SD: standard deviation
